# Taking Risks to Protect Others—Pediatric Vaccination and Moral Responsibility

**DOI:** 10.1093/phe/phad005

**Published:** 2023-05-04

**Authors:** Jessica Nihlén Fahlquist

**Affiliations:** Centre for Research Ethics and Bioethics, Uppsala University, Sweden; Department of Philosophy, Queen’s University, Canada

## Abstract

The COVID-19 pandemic during 2020–2022 raised ethical questions concerning the balance between individual autonomy and the protection of the population, vulnerable individuals and the healthcare system. Pediatric COVID-19 vaccination differs from, for example, measles vaccination in that children were not as severely affected. The main question concerning pediatric vaccination has been whether the autonomy of parents outweighs the protection of the population. When children are seen as mature enough to be granted autonomy, questions arise about whether they have the right to decline vaccination and who should make the decision when parents disagree with each other and/or the child. In this paper, I argue that children should be encouraged to not only take responsibility for themselves, but for others. The discussion of pediatric vaccination in cases where this kind of risk–benefit ratio exists extends beyond the 2020–2022 pandemic. The pandemic entailed a question that is crucial for the future of public health as a global problem, that is, to what extent children should be seen as responsible decision-makers who are capable of contributing to its management and potential solution. I conclude that society should encourage children to cultivate such responsibility, conceived as a virtue, in the context of public health.

## Introduction

The COVID-19 pandemic that started in 2020 raised several ethical questions in relation to infectious diseases and public health. One ethical issue discussed in relation to vaccination concerns the notion of autonomy—the right to make one’s own decisions. The notion is particularly relevant in relation to vaccination, that is, each individual’s capacity and right to decide for themselves whether to get vaccinated. Contributing to the protection of vulnerable individuals, maintaining healthcare systems, and possibly developing herd immunity also presuppose a sense of duty, solidarity, and responsibility for other people (Ethikrat, 2020; Nihlén Fahlquist, 2021; The Swedish National Council on Medical Ethics/SMER, 2020). By focusing on the population at large, public health measures often compromise individual autonomy, which is why ethical questions about this balance are vital (see Nihlén Fahlquist, 2019). In countries where vaccination is voluntary but strongly recommended, the infringement of autonomy is minimal. Even mandatory vaccination can be more or less coercive; for example, by imposing fees or incentives such as the right to participate in social and cultural events. Generally, countries that introduced mandatory Covid-19 vaccination have forced people to choose between the risks and benefits of having access to employment and education on one hand and remaining unvaccinated on the other. Rules regarding face masks are similar, but are less infringing in that they do not violate bodily integrity.

Children were not as severely affected by COVID-19 as adults (Zimmermann and Curtis, 2020). This finding, combined with uncertainty regarding the safety and efficacy of the vaccine and the vulnerability of children, led many to adopt a precautionary attitude to pediatric vaccination. However, during the autumn of 2021, several major public health authorities recommended pediatric vaccination based on available evidence. This recommendation concerning pediatric vaccination raises new ethical questions regarding the rights and responsibilities of parents and children.

New evidence is being collected concerning the disease as well as the efficacy and safety of the vaccine as new variants emerge. The contribution of COVID-19 vaccines to the development of herd immunity is not certain (Aschwanden, 2021). However, the pandemic has raised a general normative question about the ethics of protecting others through taking risks: what is the moral responsibility of the individual to contribute to the collective good through vaccination? This question is even more difficult when applied to pediatric vaccination. For this reason, the following discussion explores how we should conceive of pediatric vaccination and moral responsibility. The discussion is relevant not only in relation to COVID-19, but to infectious diseases and public health generally: Do children have a right and responsibility to contribute to the protection of others, and to public health? The 2020–2022 pandemic is a good illustration of these more general questions concerning the role of children in a public health context.

I argue in this paper that capable children should be encouraged to take moral responsibility not only concerning risks and benefits to themselves, but also for others. Children have an opportunity to contribute to the public good by being vaccinated, and having this opportunity can help them to develop their autonomy and ability to take moral responsibility.

After introducing the ethical debates about pediatric vaccination and children’s autonomy, I will present empirical and normative research on children as social actors with the capacity to make autonomous and responsible decisions. Finally, I will discuss how conceiving responsibility as a virtue can provide a way to understand the nature of this responsibility for children.

## The Ethics of Pediatric Vaccination in a Public Health Context

The COVID-19 pandemic hit the world hard in 2020. The rights and liberties of individuals had to be restricted to reduce fatalities and their impact on healthcare systems. The restrictions had many unintended effects on people’s lives, including interruptions to education, mental health services, cultural and social activities, and the economy. These restrictions have not only scientific and legal, but also ethical ramifications (Ethikrat, 2020; SMER, 2020). One of the most important questions is how to balance the collective good of protecting vulnerable individuals and healthcare systems, which requires a certain level of vaccine uptake, with the rights of individuals.

After the initial period of hesitation and uncertainty, major global public health authorities began in the autumn of 2021 to consider whether to recommend pediatric vaccination. In May 2021, the US Federal Drug Administration and Centers for Disease Control and Prevention (CDC, 2021a, 2021b) approved the Pfizer-BioNTech and Moderna vaccines for 12- to 15-year olds, and in October for children aged 5–11. On 14 December 2021, the state of New York began requiring children as young as five to be vaccinated. In the EU, the European Medicines Agency (EMA) initiated its evaluation for 5- to 11-year olds in November 2021 and authorized Pfizer’s vaccine before the end of 2021 (EMA, 2021).

The notions of solidarity and the responsibility of protecting others by contributing to a decrease in the spread and therefore the effects of the disease have been absent in the debate on pediatric vaccination. If the benefit to the individual child were as substantial as with measles, mumps and rubella vaccines (MMR) and human papilloma virus (HPV) vaccines, pediatric COVID-19 vaccination would be less problematic. It is more controversial to argue for pediatric vaccination for the sake of protecting others than it is to suggest this for adults, given that children are considered more vulnerable. Thus, the argument is that parents should agree to vaccination only for the sake of protecting their children. These same arguments are made for MMR vaccination—for example, the CDC states that parents should agree to the MMR vaccine in order to (a) protect their child from symptoms and (b) prevent the child from missing school and daycare (CDC, 2021a). No mention is made of preventing transmission to others. The notion of ‘the child’s best interests’ lies at the core of pediatric ethics (Kopelman 1997; Fleischman 2016; Gillam *et al.*, 2022). Against this background, it is not surprising that the main focus of pediatric vaccination is the risks and benefits to the child’s health.

In relation to COVID-19 vaccines, the CDC emphasizes that children and teens should be vaccinated to protect themselves and vulnerable family members, to be able to stay in school, and to safely participate in sports activities. The community is mentioned briefly, but the focus is on the child and close family members (CDC, 2022). In France, the National Academy of Medicine also refers to the direct individual benefit in terms of preventing severe cases and the reduction in risk to close family and schoolmates (Academie Medecine, 2021). Sweden recommended that children from the age of 12 receive the vaccine, but the only argument provided by the Public Health Agency (PHA) is the child’s own health (PHA, 2021). In 2022, the PHA ended the recommendation, stating that healthy children under the age of 17 do not need to be vaccinated (PHA, 2022). In relation to the required COVID-19 vaccination of teens, the Joint Commission on Vaccination and Immunisation (JCVI) in the United Kingdom appears to have struggled with the decision. There were tensions between the committee and the government, and the JVCI finally decided not to recommend the vaccines for healthy teenagers because the risks of the disease to them were minor and because there were marginal but severe side effects from the vaccine for some children. The committee was criticized for only taking the health benefits of children themselves into account and for acting slowly (Ahuja, 2021; Roxby, 2021). The UK’s chief medical officers did approve the vaccine for 12- to 15-year-old children (Sample, 2021). As of June 2022, the NHS webpage states that the vaccination of children stops the spread to other people, for example within schools (NHS, 2022).

## Parental Autonomy

In addition to the issues related to risks and benefits to the individual child, there are ethical questions about who has the right to make decisions concerning the child’s best interests. It is often argued that, for reasons of autonomy, parents have a right to refuse to have their children vaccinated. Autonomy is considered one of the most important values in health care, and is often described as self-governance, which is partly a competence and partly a right. That is why using the concept of autonomy in the context of a parent’s right to make decisions about their children’s health care is somewhat unusual, since the issue does not involve an individual’s right to make decisions about their own health care, but about a parent’s right to make decisions about their child’s health. A right to parental autonomy could mean different things. First, it could refer to a right for parents to choose the option that is in accordance with their own preferences. Second, it could refer to a parent’s right and/or responsibility to do what they think is in accordance with the child’s best interests. Finally, it could mean that parents have a right to be the spokesperson for the child, that is, to state and explain what the child thinks and wants to do. Arguably, the concept of parental autonomy is normatively problematic, but commonly used in discussions of health care.^1^ However, some states may not even conceive of parental decision-making in terms of a right to autonomy. One reason for this could be that the harm done by the refusal of parents to have their children vaccinated is considered to be marginal.

The concept of ‘parental autonomy’ was frequently raised in the context of vaccination even before the pandemic. Some parental organizations are highly critical of the idea that the government should force parents to vaccinate their children. For example, Wood-Harper argues that issues concerning pediatric vaccination primarily concern parents’ rights in relation to collective responsibilities for public health. These include issues such as ‘free-riding’, and potential enforcement. She argues that a balance needs to be struck and that healthcare professionals should inform parents both about risks and benefits to the child, but also implications for society at large (Wood-Harper, 2005). Similarly, Hendrix *et al.* (2016) describe the problem as involving ‘a balance between parents’ autonomy in deciding whether to immunize their children and the benefits to public health from mandating vaccines’.

Even scholars who argue that the combined interests of the community and the child outweigh the rights of the parents agree that the value of parental autonomy is relevant to the discussion. For example, Navin argues that infringements of parental autonomy are morally acceptable because parental autonomy is not as morally valuable as the autonomy of adults, and because the interests of the child and the community sometimes outweigh that of the parent’s autonomy (Navin, 2017). In the context of some vaccines, such as MMR, the focus on parental autonomy arises partly because these vaccinations have to be administered in early childhood. However, the HPV vaccine is regularly provided to girls and to some boys at the age of 11 or 12. The age of the child at the time of vaccination is clearly a relevant criterion for this discussion of balancing parental autonomy with public health.

The rights of parents are still usually considered relevant in relation to HPV vaccines. For example, Colgrove discusses a 2006 proposal to make HPV vaccine mandatory in the United States for girls entering sixth grade. He describes the critical ethical question as focusing on parental autonomy and asserts that individualistic and communitarian theories would come to different conclusions on this issue (Colgrove, 2006). Similarly, Tanne argues that the challenge is to ensure maximum vaccine uptake without overly infringing on parental rights. They point out that there are already cases where parental autonomy is overridden by what are considered the best interests of the child, for example, when parents refuse to allow their child to receive a blood transfusion for religious reasons (Tanne, 2019). However, this case is different in terms of stakes as it usually relates to a life-threatening situation that is more immediate than the effects of preventable infectious diseases.

Against this background, the role of parents in the case of vaccination could be conceptualized as entailing (1) a right to protect one’s child, and (2) a duty to protect the child. However, parents also arguably have a duty to consider the development of the child’s agency and values.

## Children’s Participation and Right to Autonomy

Although the idea of the ‘child’s best interest’ is central to pediatric ethics, the notion that children are also autonomous agents, albeit to differing degrees, has become common in recent decades. Healthcare professionals have to strike a balance in protecting the child’s interest and at the same time respecting their autonomy (Hein *et al.*, 2015a).

The primary legal issue concerns the age of majority; that is, the age at which children are considered old enough to make their own decisions. The age of the majority is different in different countries and even in different parts of one country. In Canada, it varies between 16 and 19, and legislation concerning participation in healthcare decisions also varies across provinces and may not correspond to the age of the majority. For example, in Ontario, there is no age of consent for healthcare decisions, but the age of majority is 18 (Coughlin, 2018). In most countries in the European Union, it is 18. In the United States, the age of majority is 18 in most states, but in some states, it is 19 or 21. The limit for getting a driver’s license varies between 16 and 18, but the limit for buying alcohol is 21. Similarly, in Sweden, the age of majority is 18, and the limit for getting a driver’s license is 18, but the limit for buying alcohol is 20. In other words, the age of majority is really a range—in many countries, children may make some decisions before the age of majority, while others are still prohibited.

In many countries, instead of age, capacity is considered a prerequisite for the right to make autonomous decisions in health care. The concepts of competence and capacity are often used interchangeably. There is no universal agreement regarding the age at which minors are competent to make their own decisions (Grootens-Wiegers *et al.*, 2017). There are 11-year olds with adequate capacity that are mature enough to make decisions and 16-year olds that are not.

In the United Kingdom, there is a strict line between childhood and adulthood at the age of 18, when children are considered as autonomous as adults (Great Britain, 1989), but 16- and 17-year olds are also allowed to make certain medical decisions without their parents’ approval. The child’s competence is considered more important than age. Children are also potentially considered to have the capacity to consent when their competency has been assessed, but their refusal of treatment can be overridden when it is seen as going against the child’s best interest by leading to the child’s death or serious physical or mental harm (Care Quality Commission, 2020).

Since the Gillick v West Norfolk and Wisbech Area Health Authority decision in 1986, children in the United Kingdom are considered to be legally competent to consent to medical examination and treatment if the child has achieved ‘a significant understanding and intelligence to enable/.../her to understand fully what [was] proposed’. This case concerned a 16-year-old girl’s right to receive contraceptive advice. The healthcare provider was required to make an assessment to ensure that the child understood the decision and its consequences. This so-called Gillick rule on capacity is now applicable to most treatments. However, capacity is seen as decision specific, and the rule can be overridden if a refusal of care could lead to serious injury or death, or if the minor does not fully understand the consequences of a decision to decline treatment. In other words, children are thought to be able to make some, but not all, decisions (Gillick v West Norfolk and Wisbech Area Health Authority [1986], AC 112, quoted in McLarnon 2017).

Similarly, Swedish regulations state that the age of 18 represents the strict line between childhood and adulthood. Although parents generally have a right to information about care for children under the age of 18, children are sometimes entitled to decide whether healthcare professionals are allowed to disclose information to parents. There is no specific age mentioned, but decisions are made on a case-by-case basis (1177, 2022).

In some jurisdictions, age is still important to some extent. The ‘mature minor doctrine’ has become law in certain states in the United States. For example, in Tennessee, healthcare providers are allowed in certain situations to treat minors without a parent’s consent. Children under the age of 7 have no capacity, according to the regulations. For children between 7 and 14, there is a ‘rebuttable presumption that there is no capacity and parental consent is required’. Children aged 14–18 have a ‘rebuttable presumption of capacity’ (Tennessee Department of Health, 2022).

The relationship between age, capacity and maturity is not always clear, and the terms are sometimes conflated or vague. For example, the NHS states that the Gillick rule is about capacity and not age, and that there is no lower age limit. Yet, age is sometimes mentioned, and the rule states that it would not be appropriate to apply the rule to children under the age of 13.^2^ In sum, age and capacity are two different things.

Capacity can be conceptualized in a procedural or content-focused way. Normally, capacity is assessed in a procedural manner, meaning that the content of the decisions made by patients is not evaluated. The crucial aspect is the way in which the decisions are made and not what the actual decisions involve. A patient is seen as having capacity when they ‘(a) communicate a decision, (b) understand relevant information, (c) appreciate the situation and likely consequences, and (d) reason about treatment options’ (MacKenzie and Rogers, 2013). When these criteria are fulfilled, individuals are free to make decisions, regardless of the content of the decision (Appelbaum, 2007; Mackenzie and Rogers, 2013; Ruhe *et al.*, 2016). However, according to Ruhe *et al*., there are challenges in applying a procedural account of capacity to children. This view is based on the idea of common cognitive functions and rationality, and entails exclusion of children as agents with capacity (Ruhe *et al.*, 2016).

Early discussions of children’s capacity were based on Piaget’s theories and research. He linked capacity to age, arguing that children have the same processing abilities as adults when they reach the age of 14 (Piaget, 1972). His theory has been heavily criticized for its assumptions as well as its methodology (Ruhe *et al.*, 2016). Piaget did not take into account the role of ‘social others’ and the learning context, as opposed to studying them based on what they know at a given point in time (Ruhe *et al.*, 2016).

In contrast, proponents of contextualist perspectives emphasize the role of collaboration and the sociocultural environment (Ruhe *et al.*, 2016). Similarly, feminist scholars have developed relational accounts of capacity and autonomy that emphasize how the agent’s values are affected by social relationships and by political and social context. The environment influences the development of moral agency, capacity, and autonomy. These relational (Held, 1993, 2007; Mackenzie and Stoljar, 2000; Mackenzie, 2008; Ruhe *et al.*, 2016) accounts illuminate the role and responsibility of parents and healthcare professionals, the ‘social others’, for creating an environment that is conducive to learning to make decisions and to exercise capacity (Ruhe *et al.*, 2016).

These ideas have been relevant in the context of COVID-19 vaccines. Morgan argues that 14- to 17-year olds should be allowed to make the decision on their own, and that 12- to 14-year olds should be allowed to consent to vaccination without parental approval ‘with support and facilitation from their clinicians and other trusted adult figures’ (Morgan *et al.*, 2021). According to Morgan, a policy allowing minors to be vaccinated without their parents’ consent would be a way of balancing the autonomy of minors with ‘developmental realities and parental interests’. It would use a ‘sliding scale of decision-making authority, granting greater autonomy to minors as they age while also considering the risks and benefits of vaccination’. As described above, this is the case in some jurisdictions where decision-making is based on capacity as opposed to age of consent.

There are scholars who do not consider children to be autonomous. Giubilini *et al.* (2020) argue that if and when immunization of children benefits the elderly, they should be vaccinated even if the risk is not zero and the benefit not as great as it is for the elderly. Their argument primarily concerns the prioritization of resources, mandatory vaccination and whether it is permissible to use children as a means to protect vulnerable people. They mention autonomy, but only dismiss it as irrelevant. They argue that vaccinating children ‘cannot meaningfully infringe on their autonomy simply because children do not have the relevant capacity for autonomous decisions’ (Giubilini *et al.*, 2020). They argue that since we do not ‘normally’ treat children as autonomous, we should not do it in the case of vaccination. This is an unusual argument. As described above, there is an ongoing discussion about when children should be considered capable of autonomously making medical decisions. There are also laws in most countries granting children substantial rights to autonomy. The only example the authors give in which children’s potential right to autonomy is overridden is in the case of bone marrow transplants. However, there are numerous other examples where children’s right to autonomy is accepted, such as in decisions concerning birth control pills.

## Children as Actors

Since children need protection due to their vulnerability, it is understandable that parents want to reduce their children’s exposure to perceived risks by, for example, not accepting a new vaccine.

However, focusing on protection instead of allowing children to develop their own decision-making capacity could be seen as a misconceived trade-off of risk. Psychological studies indicate that parents’ desire to protect their children sometimes goes too far (see James and James, 2001; Zeiher, 2001; Prout, 2005). Children whose parents provide them with opportunities for self-regulation and participation in decision-making are more likely to develop self-worth and avoid depression (Prout and James 2015).

To provide support for the child in relation to parents, several social science disciplines conceptualize children as social actors, which is a highly relevant approach in the context of pediatric vaccination. Children as social actors act in ways that potentially have an impact on other people and are capable of making reasonable decisions (Pritchard, 1991, 1996). These ideas are also supported by the United Nations Convention on the Rights of the Child (CRC, 2022) as well as many national jurisdictions.

Questions of autonomy and children often arise in relation to their right to make decisions concerning healthcare involving risks and benefits to themselves. However, in some cases, children are considered capable to make decisions that potentially impact others as well. One example is participation in clinical trials. In one study, children said they participated in clinical trials to help both themselves and others. In one study, children from the age of 10 stated that the reason for participating in a clinical trial was to benefit or help others and even in some cases help advance knowledge (Hein *et al.*, 2015b).

Against this background, it should be possible to see that some children are capable of making decisions concerning the effect of their actions on vulnerable people and the population at large. The capacity to make decisions concerning one’s own health may in practice, and in many cases, overlap with the capacity to make decisions that affect others. However, this is not necessarily and universally the case. Furthermore, the normative question of whether children should be allowed to make decisions that affect others, but not directly themselves, remains.

Since there is always a power balance between parents and children, there is always the risk that parents will have the final say, and it is difficult to know to what extent a decision is made by the parent or the child. The power balance only becomes apparent to others when parents disagree with each other or when a mature child disagrees with the parent to the extent that the dispute becomes a legal matter (Weichel, 2021).

## Children and Moral Responsibility for the Protection of Others

The capacity and right to make decisions are usually connected to notions of moral responsibility. When making decisions concerning oneself—and even more so when those decisions affect others—it is reasonable to ascribe some degree of moral responsibility to the actor. For this reason, one crucial ethical aspect of vaccination discussed in relation to adults is to what extent individuals have a moral responsibility to get vaccinated to contribute to public health and the protection of vulnerable individuals (Ethikrat, 2020; SMER, 2020; Nihlén Fahlquist, 2021).

In many ways, public health issues such as these resemble other collective problems, for example, climate change. What we do as individuals in the context of a pandemic affects others in a more direct way than, for example, receiving cancer treatment or taking medications. Furthermore, these problems are more complex today than at any time in history. What we do in one part of the world potentially affects others far away. This complex nature of contemporary collective problems calls for a stronger sense of responsibility (see Young, 2006).

This kind of societal problem is not likely to be solved or eradicated any time soon. For this reason, there is an urgent need to involve children as future decision-makers. In relation to climate change, this is recognized through educational requirements (see Trott, 2019). Whereas some scholars argue that children should only be taught to perform the ‘right actions’, others argue that the goal should be to help people of all ages become more aware of the world around them and of the need for individual as well as social change, and to ‘develop critical thinking and capacity to address rapid change and uncertainty’ (Bangay and Blum, 2010: 8). Public health problems like pandemics and antibiotic resistance should lead to the same efforts to engage children to think and act in ways that contribute to solutions as does climate change. This raises the question of what concept of responsibility is most relevant in the context of children.

Responsibility is a multifaceted and complex concept and can refer to tasks, roles, obligations and accountability. In this context, seeing responsibility as a character trait that is developed over time, with practice and the help of role models, highlights the importance of helping children from a young age to gradually start to see themselves as having an important role to play in society.

The concept of responsibility as a virtue dovetails with studies that illuminate the social and moral agency of children and their gradual development of cognitive and moral capacities. In contrast to consequentialist ethics and deontological ethics, which are focused on actions and rules about right and wrong, virtue-ethical theories illuminate the importance of a person’s character or dispositions to act in morally excellent ways.

## Historical Perspectives

There are different kinds of virtue-ethical theories. First, Neo-Aristotelian virtue ethics is based on the Aristotelian notion of eudaimonia, that is, flourishing, which is thought to be the purpose of life. This state is achieved by developing both the virtues and external goods; for example, wealth and friends, and the purpose of life is to live well through exercising the virtues. As human beings, we are capable of rational thinking, and the virtues are informed by phronesis, that is, practical reasoning. We are not born possessing virtues, but we have the capacity to become virtuous (Snow, 2020). In contrast, agent-based virtue ethics focus on the agents themselves and their motivations, emotions and dispositional qualities (see Slote, 2001, 2020; Zagzebski, 2004). Third, target-based virtue emphasizes a ‘good quality of character, more specifically a disposition to respond to, or acknowledge, items within its field or fields in an excellent or good enough way’ (Swanton, 2021).

All of these theories are relevant in the context of children. For the purposes of this paper, it is not necessary to go into the complexities of the different theories. All of these have made important contributions to ethical discussions. However, it would be useful to emphasize the most important aspects in the context of children, vaccination and public health. First, human beings are more complex than their actions and it is not reasonable to assess decisions in isolation from the people making them and the context in which those decisions are made. Second, although we have different backgrounds, conditions and circumstances, most of us can, and do, develop a moral compass through experience and reflection. Third, our development and cultivation can be facilitated or obstructed by other people, structures, and institutions (MacKay, 2021; Nihlén Fahlquist, 2022).

Long-term societal challenges with extensive uncertainty call for a sense of personal responsibility that transcends rule-abidance (Ethikrat, 2020; Nihlén Fahlquist, 2021). Rules are important but not adequate to deal with such profound problems. Instead of merely focusing on so-called ‘downstream interventions’ like rules and regulations, ‘upstream interventions’ are needed, where the focus is on cultivating an environment that creates the necessary behavioral changes (Verplanken and Wood, 2006; Maio *et al.*, 2007).

Being a responsible person in this sense entails engaging with others even when it requires sacrifices (van Hooft, 2006). As argued by Williams, it represents a ‘readiness to respond to a plurality of normative demands’ (Williams, 2008). Living in contemporary societies is more complex in many ways than living in premodern times. That is why the notion of plurality is important. Taking responsibility in this way, navigating this complex world requires creativity, attention, and a degree of discretion due to uncertainty concerning the future and the extremely fast pace of change, accompanied by many conflicting demands, in society today.

The pandemic requires figuring out how to care for the elderly without compromising their physical health and how to keep children indoors without damaging their physical and mental health. In contemporary societies, the individual must be trusted to exercise some degree of discretion to navigate these demands. Along the same lines as Williams’ description of responsibility as a virtue, Young describes the new sense of responsibility required by the modern world as deriving from ‘belonging together with others in a system of interdependent processes of cooperation and competition through which we seek benefits and aim to realize projects’. We may not be able to see the results or know the exact causality or outcome of our own individual actions, but as part of a scheme of social cooperation aiming to achieve some good together, we all legitimately expect justice toward ourselves and ought to develop a sense of responsibility toward others (Young, 2006).

It can be argued that virtues are not relevant to public health, which essentially is utilitarian in that it aims to maximize the health of the population at large. Arguably, utilitarianism has limitations in terms, for example, of which consequences should be considered and how health outcomes should be measured. However, it can also be argued that decisions about public health must at some level be based on a collective calculation. Virtue ethics could be considered inapplicable because of its lack of action-guiding, which is a common critique against virtue ethics as an ethical theory. However, the number of virtue-ethical accounts of public health is increasing (Rozier, 2016; Nihlén Fahlquist, 2019; MacKay, 2021). While a utilitarian focus may be necessary at the level of societal decision-making, virtue-ethics adds important aspects to the utilitarian perspective. When dealing with complex and profound societal challenges that cannot be easily managed, long-term changes in habits and ways of thinking are necessary. Climate change, environmental and social sustainability, antibiotic resistance, and pandemics are examples of such problems. Interestingly, Jamieson argues that utilitarians should embrace virtue ethics when addressing the environment because of the substantial changes in behavior that are required in order to create a sustainable society (Jamieson, 2007). Kristjánsson discusses the role of character development from an early age, facilitated by education. He argues that virtues are reason responsive and educable and should be facilitated and taught in schools (Kristjánsson, 2015).

## Obligations or Ideals?

One further question that arises is to what extent it should be obligatory for children to get vaccinated. According to some theories, there are actions that are not obligatory, but rather praiseworthy, morally admirable or heroic. These acts are so-called ‘supererogatory acts’. Urmson (1958) criticizes conventional ethical theories that, according to him, do not take the latter into account. What it means for an act to be supererogatory, and whether the concept is intelligible or useful, is a subject for discussion. The notion of supererogatory actions has been seen as incompatible with the virtue-ethical concept of the mean. Aristotle described a virtue as an intermediate condition, a mean between extremes. Excess and deficiency are both vicious and should be avoided, and the virtuous thing to do is the intermediate. That would imply that if we go beyond the intermediate, we act excessively, which would be vicious. If this is true, there can be no virtue that goes beyond the ‘right thing to do’, whose omission would not be vicious. One either acts virtuously or viciously (Stangl, 2016). However, as several philosophers argue, if virtues are targets, then it is plausible to think that there is more than one way to hit the target, and, consequently, that there is more than one way to be virtuous. As Stangl argues, if two people pass a homeless person and one of them gives them 20 € and the other one gives them 10 €, both have been generous but to differing degrees (Stangl, 2016).

Recently, virtue-ethicists have started to discuss how it is possible to accommodate such notions within a virtue-ethical perspective (Vaccarezza, 2019). Vacarezza defines a supererogatory action within a virtue-ethical framework as follows:

Supererogatory actions are the best overall actions that are not required of an ordinarily virtuous agent, facing an extremely difficult circumstance, where ‘extremely difficult’ means requiring a heroic degree of virtue. (Vaccarezza, 2019).

Stangl’s suggests that an act is supererogative ‘if and only if it is overall virtuous and either (a) the omission of an overall virtuous action in that situation would not be overall vicious or (b) there is some overall virtuous action that is less virtuous than it and whose performance in its place would not be overall vicious’.

Without adhering to one definition, the most important notion in this context is that virtues can exist in degrees. The seriousness of deciding whether or not to be vaccinated is likely to be perceived differently by different individuals, children as well as adults. As Vaccarezza argues, what counts as supererogatory cannot be predetermined for every agent. It is dependent on the degree of virtue one has achieved (Vaccarezza, 2019).

Ideal character traits cannot be immediately and universally expected by everyone. Virtues are developed over a lifetime, and it is reasonable to think of virtues as a matter of degrees: that is, that an agent can be more or less virtuous and that they ideally become more virtuous as they go through life. It is not reasonable to expect children to be fully virtuous in relation to these societal problems without practice, support and experience. Responsibility conceptualized as a virtue is about learning to respond to a plurality of normative demands (Williams, 2008), but it is also about weighing different normative demands. What starts out being supererogatory for young children may then become what is expected of a fully virtuous adult.

## Conclusion

Discussions of pediatric vaccination and autonomy focus on the right to decline vaccination if parents or capable children consider the risks to outweigh the benefits to themselves. But the COVID-19 pandemic brought questions regarding responsibility for protecting others against harm to the fore. This aspect of the discussion has not adequately involved children. While the disease is not as severe a threat to children as other infectious diseases such as measles, the value of protecting others in this case is more salient. The discussion of pediatric vaccination in cases where this kind of risk–benefit ratio exists extends beyond the 2020–2022 pandemic. The pandemic entailed a question that is crucial for the future of public health as a global problem, that is, to what extent children should be seen as responsible decision-makers who are capable of contributing to its management and potential solution.

As discussed in this paper, many jurisdictions treat children of various ages as autonomous, to the extent that they are considered capable. These laws commonly refer to children’s right to participate in decision-making concerning their own health. Against the background of arguments supporting children as social actors, it is reasonable to also consider children’s ability to take responsibility for others. Vaccination should be seen as an opportunity for children to develop a sense of responsibility. Parents, schools, healthcare staff and public health agencies can make efforts to include children in the ethical debate on public health. These discussions should not merely focus on the risks and benefits of the vaccine to the children themselves, but also on how their actions affect others and how they can contribute to addressing the problem. Responsibility as a virtue in the context of public health should be considered a character trait that develops over time with experience and support. However, this requires a delicate balancing act, and the vaccination of children should not be used purely as means to protect others. Education and communication concerning ethical arguments on vaccination should be used to facilitate an understanding of the effects of individual actions on society at large. Whether and to what extent this is a form of pressure needs to be further discussed.

## Footnotes

1 I would like to thank an anonymous reviewer for pointing this out.

2 I would like to thank an anonymous reviewer for pointing out this inconsistency.
